# Anti-Aβ Autoantibodies in Amyloid Related Imaging Abnormalities (ARIA): Candidate Biomarker for Immunotherapy in Alzheimer’s Disease and Cerebral Amyloid Angiopathy

**DOI:** 10.3389/fneur.2015.00207

**Published:** 2015-09-25

**Authors:** Jacopo C. DiFrancesco, Martina Longoni, Fabrizio Piazza

**Affiliations:** ^1^School of Medicine, Milan Center for Neuroscience (NeuroMi), University of Milano-Bicocca, Monza, Italy; ^2^The Inflammatory Cerebral Amyloid Angiopathy and Alzheimer’s Disease βiomarkers (iCAβ) International Network, Monza, Italy; ^3^The iCAβ-ITALY Study Group of the Italian Society for the Study of Dementia (SINdem), Monza, Italy

**Keywords:** cerebral amyloid angiopathy related inflammation, *i*CAβ International Network, Alzheimer’s disease, cerebrospinal fluid biomarker, amyloid-related imaging abnormalities, Aβ disease modifying therapies, immunotherapy, anti-amyloid antibody clinical trials

## Abstract

Amyloid-related imaging abnormalities (ARIA) represent the major severe side effect of amyloid-beta (Aβ) immunotherapy for Alzheimer’s disease (AD). Early biomarkers of ARIA represent an important challenge to ensure safe and beneficial effects of immunotherapies, given that different promising clinical trials in prodromal and subjects at risk for AD are underway. The recent demonstration that cerebrospinal fluid (CSF) anti-Aβ autoantibodies play a key role in the development of the ARIA-like events characterizing cerebral amyloid angiopathy-related inflammation generated great interest in the field of immunotherapy. Herein, we critically review the growing body of evidence supporting the monitoring of CSF anti-Aβ autoantibody as a promising candidate biomarker for ARIA in clinical trials.

## Introduction

Biomarkers for the stratification, follow-up and monitoring of the safe and effective therapeutic response of amyloid-beta (Aβ) disease-modifying therapies (DMT) represent a research priority in Alzheimer’s disease (AD) (1).

**Figure 1:**
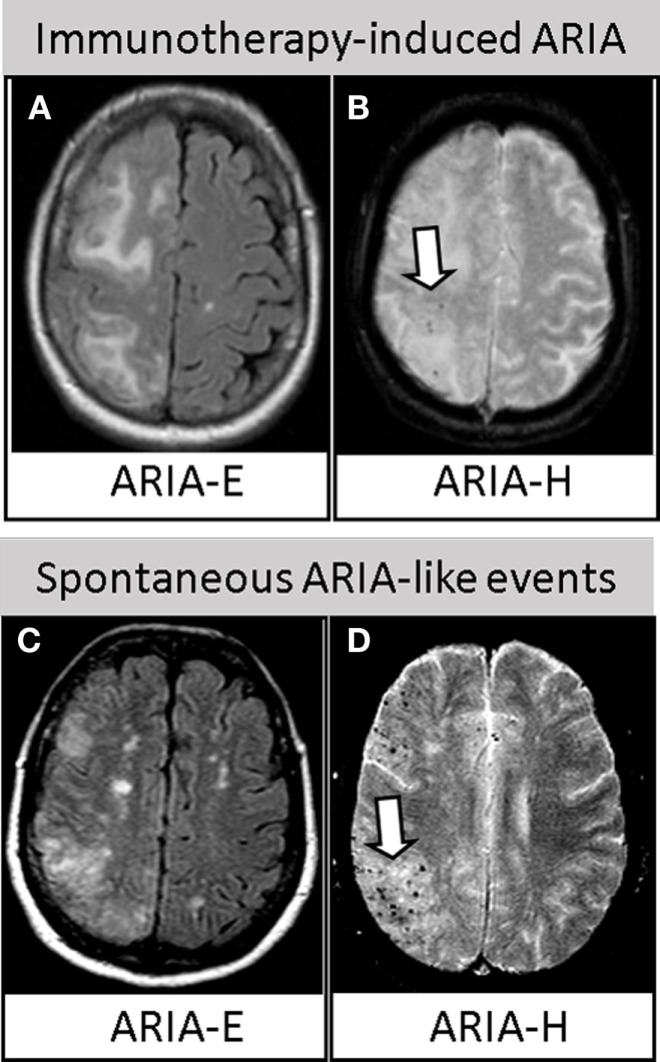
**Similarities between immunotherapy-induced ARIA and spontaneous ARIA-like events in CAA-ri**. *Upper row*. Axial brain MRI revealing ARIA-E **(A)** and ARIA-H [**(B)**, arrow] in one AD patient treated with bapineuzumab. Reproduced with permission from Ref. (3). *Lower row*. Axial brain MRI revealing spontaneous ARIA-E **(C)** and ARIA-H [**(D)**, arrow] occurring in one CAA-ri patient from the “The inflammatory Cerebral Amyloid Angiopathy and Alzheimer’s disease βiomarkers (*i*CAβ) International Network (20, 50).” Images **(A,C)** represent FLAIR-MRI sequences; **(B,D)** represent T2*-GRE sequences.

Immunotherapy trials, in particular, have underlined the urgent need of safety biomarkers to avoid, or at least enable the early detection of the severe side effects of treatment termed amyloid-related imaging abnormalities (ARIA) (2). There are two types of ARIA: ARIA-E, characterized by the magnetic resonance imaging (MRI) evidence of vasogenic edema (VE) and/or sulcal effusion on fluid-attenuated inversion recovery (FLAIR), as hallmarks of inflammation at the level of the affected vessels; and ARIA-H, characterized by signal of hemosiderin deposits involving microhemorrhages (MHs) and superficial siderosis on T2*-weighted gradient echo (T2*-GRE) or susceptibility-weighted imaging (SWI), as hallmarks of cerebral amyloid angiopathy (CAA) (3) (Figures 1A,B).

Even if the acronym ARIA was initially referred to specifically describe the MRI abnormalities of bapineuzumab (2, 4–6), the first monoclonal antibody employed in clinical trial, the term is currently used to define the clinical–radiological side effects subsequently reported with almost all the immunotherapy strategies tested (7–12).

Today, no early biomarker able to predict the incipient occurrence of an ARIA has been already included in clinical trials. However, the current FDA guidelines for enrolling patients in studies assessing DMT require MRI evaluation, recommending to exclude patients with ≥5 MHs and with any evidence of superficial siderosis or prior parenchymal hemorrhage (3, 6). Nevertheless, MHs on MRI are relatively non-specific, reflecting a variety of pathologic conditions. MRI could thus be particularly helpful for the detection of the acute/subacute course of ARIA, but it could fail to predict patients at high risk to develop incipient occurrence of these events, both at the baseline and during the therapeutic follow-up (13–18).

The ARIA issue recently generated increasing interest after the very promising data for the Phase 1b study of aducanumab (NCT01677572) were presented at the 12^th^ AD/PD Meeting in Nice (19) and at the Alzheimer’s Association International Conference (AAIC 2015) in Washington (12). This drug demonstrated a statistically significant cognitive improvement in patients with prodromal or mild AD, together with a dose- and time-dependent reduction of deposited Aβ on amyloid-PET. Aducanumab, however, revealed an incidence of immunotherapy-related ARIA in the 55% of patients, particularly in the high-dose and *APOE*ϵ*4* carriers arm, associated with a 35% of ARIA drop-outs due to the development of these side effects (19).

The recent discovery that ARIA-like events in CAA-related inflammation (CAA-ri) are mediated by increased anti-Aβ autoantibodies in the CSF, has sensibly increased the understanding of the etiological mechanisms of ARIA. CAA-ri has thus been proposed as a human spontaneous model of the drug-induced ARIA in AD (15–17).

Starting from this background, in this review we critically discuss the growing body of evidence supporting the dosage of CSF anti-Aβ autoantibody as a promising candidate biomarkers for ARIA in clinical trials (13, 15–17, 20, 21).

## Immunotherapy-Induced ARIA

Trials in AD and natural history studies have suggested that the following all contribute to the development of ARIA: 1) the severity of Aβ deposition (e.g., greater in advanced stages of the disease), 2) the degree of CAA in an already impaired vasculature, 3) the *APOE*ϵ*4* allele dose, and 4) the dose of drug administered.

In human clinical trials, although the mechanisms leading to ARIA are not yet fully elucidated, it is well demonstrated that increased drug dosage clearly augments the risk to develop ARIA (4, 11, 12, 19). Another interesting aspect is that *APOE*ϵ*4* carriers, with higher parenchymal and vascular Aβ load, are more vulnerable to ARIA, due to the larger antibody-enhancement shift in Aβ. Consistently, the analyses of the two phase III trials of bapineuzumab showed a greater incidence of ARIA in association with the number of *APOE*ϵ*4* alleles, increasing from 11.4% in *APOE*ϵ*4* heterozygotes to 27.3% in *APOE*ϵ*4* homozygotes. Interestingly, *APOE*ϵ*4* carriers represented the well-responder group of patients, showing a dose-related reduction of CSF tau and phospho-tau and a decreased rate of Aβ accumulation on amyloid-PET after treatment with bapineuzumab (4, 6, 22), gantenerumab (11), and aducanumab (12, 19).

A retrospective revision of all MRI scans of patients included in the bapineuzumab trials identified an even larger number of ARIA cases (35%) than those previously described (17%), in line with the recent data emerged for aducanumab (55%). Particularly, ARIA-E were reported as the most common abnormalities, while nearly half of the ARIA-E positive cases also developed ARIA-H, often colocalized in the same brain regions. In addition, it has been shown that these abnormalities tended to occur early in the course of treatment, with most occurring between the first and third infusion. ARIA can present with relevant neurological signs, characterized by headache, confusion, and neuropsychiatric symptoms. Patients, however, may also experience mildly symptomatic or asymptomatic ARIA, rapidly resolving with the discontinuation of treatment (3, 4, 6, 11, 12, 19).

Of note, ARIA have always been reported to be paradoxically more represented in patients treated at the higher, but more effective, dosages of the administered therapeutic antibody (2, 4, 6–12, 19), thus dramatically increasing the interest in biomarkers for understanding, predicting, and monitoring these potential hazards (14, 15, 17).

## Spontaneous ARIA-Like Events

In 2013, the discovery that the typical MRI findings of VE (ARIA-E) and multiple area of MHs and/or superficial siderosis (ARIA-H) characterizing the acute phase of CAA-ri represent a variation of drug-induced ARIA has generated great interest in the field of immunotherapy (16). Following this first evidence, several subsequent studies have clearly confirmed the clinical and radiological similarities. CAA-ri is characterized by symptomatic or mildly symptomatic acute/subacute neurological signs, mainly headache, mental confusion, psychiatric symptoms, dizziness, and focal signs. Moreover, like in AD trials, the MRI features are represented by asymmetrical and bilateral VE involving the posterior cortical/subcortical white matter, and by diffuse MHs or signs of cortical superficial siderosis (Figures 1C,D). Additionally, as for immunotherapy-induced ARIA, the *APOE4* genotype is overrepresented in CAA-ri patients (16, 23–30). Another interesting finding is that CAA-ri patients are typically very well responsive to immunosuppressive therapy if diagnosed and medicated promptly, rarely reporting the occurrence of successive relapses (20).

Of note, spontaneous ARIA-like events have been recently identified in prodromal (21, 31) and established AD (32), and in one case, the development of ARIA has been reported as a possible trigger for rapidly progressive dementia (21).

Interestingly, spontaneous ARIA and CAA-ri have been also described in familial forms of AD (FAD), i.e., in AβPP duplication carriers (33), in presenilin 1-associated FAD (I202F *PSEN1* mutation) (34), and in two siblings carrying the P284S *PSEN1* mutation (35). Recognition that ARIA may arise spontaneously during the course of FAD is a particular timely and important observation that further reinforces the parallelism between iatrogenic and spontaneous ARIA, given the immunotherapy trials for FAD underway.

## Anti-Aβ Antibodies as Biomarker for ARIA

The lack of reliable techniques for the detection of anti-Aβ autoantibodies have so far led to contradictory results, showing a reduced (36–38), partially modified (39), unchanged (40, 41), or even increased amount in AD patients (42, 43). A possible explanation is that these studies were conducted in plasma or serum, while their CSF levels have never been clearly explored before.

The recent development of an ultra-sensitive technique able to detect the very low concentration of anti-Aβ autoantibodies in the human CSF has sensibly increased the understanding of their physio-pathological functions (16). CSF anti-Aβ autoantibodies have been demonstrated to play a key role in the etiopathogenesis of ARIA-like in CAA-ri and, today, CAA-ri is widely accepted as a human spontaneous model of the therapeutic-induced ARIA (15–17).

First, like in immunotherapy, the acute phase of CAA-ri is characterized by a specific immune reaction mediated by an increased amount of autologous CSF antibodies against the perivascular deposited Aβ typical of CAA (13, 16, 21, 44–46). Although observed in a single case study, autoantibodies have been found to be intrathecally produced and specifically increased only in CSF, while no changes has been found in the plasma, thus reflecting the immune/inflammatory mechanisms restricted to the brain (13). However, considering the less invasive procedure compared to CSF, further investigations in a larger population will be of certain interest. Second, the temporal relationship between anti-Aβ autoantibody levels and clinical and radiological improvement of CAA-ri strongly supports they are a specific trait of the VE and MHs processes (ARIA-like events) (13, 16, 21, 46). Third, like in AD-treated patients, the increased CSF level of Aβ40 and Aβ42, the decreased amyloid-PET uptake, and the higher amounts of anti-Aβ autoantibodies indicate a transient massive drainage of Aβ from the brain and vascular deposits to its soluble forms (18, 20, 31, 47). Furthermore, in line with data from passive immunization (8, 48), a reduction of both autoantibodies and neurodegenerative markers tau and P-tau in the CSF has also been demonstrated following the clinical and radiological remission of the acute phase of the disease (16, 21). Fourth, the levels of anti-Aβ autoantibodies specifically discriminate CAA-ri from sporadic CAA without inflammation, other non-CAA inflammatory and autoimmune disorders or healthy controls (13, 16, 20, 21). Fifth, anti-Aβ autoantibodies have been suggested as a possible early predictor of CAA-ri recurrence (20).

Such insights have definitively pointed out the dosage of CSF anti-Aβ autoantibodies as a very promising candidate biomarker for the diagnosis, monitoring and management of ARIA. Currently, although the validation of cut-offs for clinical diagnostic purposes is still ongoing, the dosage of CSF anti-Aβ autoantibodies in CAA-ri is already accepted as a valid support in clinical practice (49).

## Future Directions in Immunotherapy Trials

The recognition that CSF anti-Aβ autoantibodies represent a valid biomarker in the diagnosis of CAA-ri paves the way for new avenues in immunotherapy of AD. Studies aiming to quantify the amount of naturally occurring anti-Aβ autoantibodies in AD patients enrolled in clinical trials should thus be taken in serious consideration (49).

The inflammatory Cerebral Amyloid Angiopathy and Alzheimer’s disease βiomarkers (*i*CAβ) International Network, a World-Wide Consortium aimed to the discovery and validation of biomarkers of ARIA in the largest cohort of CAA-ri today available, represents a leading authority in the field (50).

Here is an example of the different critical information that may derive by the measurement of CSF anti-Aβ autoantibodies as promising candidate biomarker for ARIA.

### Patient engagement biomarker

The baseline level of CSF anti-Aβ autoantibodies in AD and healthy subjects is currently unknown. A key area for future studies will be to explore the levels and time course of CSF anti-Aβ autoantibodies at the baseline (before treatment) and during immunotherapy. Of note, the high prevalence of *APOE*ϵ*4* carriers and the co-localization of MHs and VE in CAA-ri (20) further strength the indication to dose anti-Aβ autoantibodies as a potential biomarker to identify those patients at higher risk of ARIA. Notably, these findings will be of direct relevance also for CAA, since the first phase I immunotherapy trial (ponezumab) for sporadic CAA has recently been launched (NCT01821118).

### Drug tailoring biomarker

The monitoring of CSF anti-Aβ antibodies (both therapeutically administered and naturally produced) may allow personalizing treatment for a greater clinical effect, minimizing the occurrence of ARIA side effects, in order to maintain a putative “therapeutic window” for the safe clearance of vascular Aβ. This may be particularly true for patients at high risk for ARIA (*APOE*ϵ*4* carriers and/or high CSF autoantibody at the baseline). This may also explain the lack of efficacy of previous immunotherapy trials compared to aducanumab. The dosage of the therapeutic antibody has often been limited due to the concerns of ARIA side-effect, leading to the exclusion of patients from the opportunity to be treated and the continuous adjustment of the therapeutic protocols.

### Safety prediction and drug engagement biomarker

The identification of cut-off for ARIA-like events has been demonstrated to be a valid diagnostic biomarker in CAA-ri. The measurement of the CSF anti-Aβ antibodies titer in patients developing ARIA could allow establishing similar reference values for the prediction or, at least, the early diagnosis of these events during immunotherapy of AD. This could permit the management of treatment, e.g., reducing the dosage or delaying further infusions in patients at risk for ARIA. This could be particularly important between the second and third drug administration, since the majority of ARIA have been reported during this period (2, 4, 6, 8). Moreover, the monitoring of CSF anti-Aβ antibodies, together with the proof of reduced Aβ accumulation on amyloid-PET (11, 12, 22) and the increased level of CSF Aβ, could be proposed as an additional biomarker to monitor drug efficacy and for a better interpretation of the trial outcomes.

### Biomarker for ARIA remission at follow-up

In the case of ARIA occurrence, an early diagnosis will allow a prompt medication, e.g., steroid administration, thus avoiding the exclusion of these patients from trials. Furthermore, the return of CSF anti-Aβ antibodies below a putative cut-off level (still to be established) could help clinicians in confirming the effective remission of ARIA, as efficiently demonstrated in CAA-ri (16).

## Conclusions

In the last decade, ARIA have severely limited the development of DMT. The validation of anti-Aβ autoantibodies biomarker for the monitoring and prediction of ARIA could have critical implications to avoid the occurrence of these serious side-effects. Anti-Aβ autoantibodies may offer a unique possibility to explore the relationships between Aβ clearance and the outcomes of clinical trials, increasing the chances for developing innovative DMT.

The monitoring of CSF anti-Aβ autoantibodies could help personalized treatment. The stratification of patients based on the risk to develop ARIA could allow their allocation in the right dosage arm in order to obtain the best therapeutic window for each specific treatment and study. Of note, since we are moving to larger and longer prevention trials in prodromal and subjects at risk for AD, based on the selective enrollment of patients with positive CSF and/or amyloid-PET uptake (Table 1), this is a particularly timely issue that could potentially increase the risk to incur in the same side effects (ARIA) previously reported. Noteworthy, the enthusiasm for the very promising perspectives emerging for aducanumab (NCT01677572) and gantenerumab (NCT02051608 and NCT01760005) may be affected by patient complains related to the high risk of ARIA, thus reducing their feeling in the treatment. Without effective biomarkers we will have the consequence of further unacceptable delays in finding a cure for these devastating diseases.

**Table 1:** **Use of CSF and amyloid-PET biomarkers in current immunotherapy trials of Alzheimer’s disease and cerebral amyloid angiopathy (update – September 2015)**.

Drug	Condition	Trial phase	Biomarker use		Clinical trial government identifier
			CSF	Amyloid PET	
Solanezumab (A4 study, Expedition3, ExpeditionEXT)	Asymptomatic AD or mild to moderate AD	III	Outcome evaluation	Inclusion and outcome evaluations	NCT02008357
					NCT01900665
					NCT01127633

Gantenerumab	Prodromal or mild AD	III	Inclusion and outcome evaluations	Inclusion and outcome evaluations	NCT02051608
					NCT01224106

Gantenerumab and Solanezumab (DIAN-TU)	Autosomal dominant AD	II	Inclusion and outcome evaluation	Inclusion and outcome evaluation	NCT01760005
		III	

Crenezumab	Prodromal autosomal dominant AD kindred or mild to moderate AD	II	Outcome evaluations	Inclusion and outcome evaluations	NCT01998841
					NCT02353598

Aducanumab (BIIB037 study, ENGAGE, EMERGE)	Prodromal or mild AD	I	–	Inclusion and outcome evaluations	NCT01677572
		III			NCT02484547
					NCT02477800

Ponezumab	Cerebral amyloid angiopathy	II	–	–	NCT01821118

BAN2401	Early AD	II	–	Inclusion and outcome evaluations	NCT01767311

*CSF, cerebrospinal fluid; AD, Alzheimer’s disease*.

Biomarkers for ARIA will also improve our understanding on the mechanisms of action and drug efficacy of immunotherapies, i.e., the decreased Aβ load observed on amyloid-PET (8, 11, 12, 22) and the associated positive effects on downstream markers of neurodegeneration (11, 12, 48).

Although the study of CSF and/or imaging biomarkers for ARIA is matter of current active investigation (50), as highlighted in this review, more research is obviously needed.

The validation of biomarkers for ARIA will necessarily imply a multidisciplinary approach and the more strict collaboration between pharmaceutical companies leading immunotherapy trials, clinicians, basic researchers from academy, research societies and regulatory authorities.

In the near future, the comprehension of the physiopathological mechanisms of ARIA and the discovery of early biomarkers will represent an important challenge in order to ensure safe and beneficial effects of immunotherapy (16, 17, 49). Therapeutic implications for CSF anti-Aβ autoantibodies biomarker would be of immediate application, representing a unique benefit of DMT efficacy compared to other more expensive techniques such as amyloid-PET. CSF withdrawal is in effect a common and minimally invasive diagnostic procedure widely used in clinical trials (Table 1). The opportunity to implement this biomarker should thus be taken in serious consideration, particularly in the suspicious of ARIA.

## Author Contributions

FP contributed in the conception, design, and drafting of the work. JCD and ML contributed in the drafting and critical revision. All the Authors approved the final version of the work.

## Conflict of Interest Statement

Jacopo C. DiFrancesco and Martina Longoni report no conflicts of interest. Fabrizio Piazza is the inventor of the patent “A Method And A Kit For The Detection Of Anti-Beta Amyloid Antibodies” for which the University of Milano-Bicocca is the only applicant and owner. Fabrizio Piazza does not have any commercial or financial relationship with the patent.
